# Gastrointestinal Tract Cancers, an Increasing Burden of the Modern Era: Epidemiology and Prevention

**DOI:** 10.3390/cancers15184634

**Published:** 2023-09-19

**Authors:** Stefano Kayali, Elisa Marabotto, Edoardo Giannini

**Affiliations:** 1 Gastroenterology Unit, Department of Internal Medicine, IRCCS Ospedale Policlinico San Martino, University of Genoa, 16132 Genoa, Italy; elisa.marabotto@unige.it (E.M.); egiannini@unige.it (E.G.); 2 Department of Medicine and Surgery, University of Parma, 43121 Parma, Italy

Gastrointestinal tract cancers, including oral, oesophageal, stomach, liver, pancreas, and colorectal cancers, represent a significant worldwide health concern [1]. These tumors collectively represent a considerable portion of all global cancers, accounting for around one quarter of oncologic diagnoses and almost one third of cancer-related deaths [2]. While some risk factors such as smoking, diet, lifestyle and alcohol consumption are shared, each tumor exhibits unique epidemiological characteristics and factors contributing to its development [1]. The role of primary risk factors is embodied both by chronic and modifiable conditions. These adjustable variables offer a field where extensive possibilities of intervention lie and where research should focus in order to optimize prevention strategies against these malignancies.

The epidemiology of gastroenterological neoplasms is undergoing changes due to modifications in the prevalence of their risk factors, leading to variations in their incidence. It is foreseen that the number of new cases and mortality from these cancers will rise by 58% and 73%, respectively, over the next 20 years, reaching alarming numbers of 7.5 million and 5.6 million deaths per year [2]. Therefore, addressing patient-tailored risk assessment and prevention strategies is imperative to contrast this increasing burden.

In this Special Issue, we present a collection of articles that focus on recent advancements in risk assessment and prevention modalities for gastrointestinal and liver cancers. These studies highlight various preventative aspects and the consequent need for targeted interventions.

Primary prevention plays a fundamental role in the modification of cancer epidemiology. Hepatitis B vaccination and lifestyle modifications have demonstrated their efficacy in reducing the risk of liver cancer, as shown by a study on risk factors in patients with metabolic-associated fatty liver disease, while the impact of care pathways, delays, and socio-spatial determinants on pancreatic adenocarcinoma development was explored in a study carried out in the PANDAURA cohort [3,4]. This study, which involved 76 French centres and over 500 patients, confirmed that the surgical management of this disease should be prerogative of highly specialized, high-volume centres, given that they are the sole institutions capable of ensuring a longer post-surgical survival. Interestingly, it has also brought to the attention of the scientific community the importance of organizing territorial medicine. An integrated approach to general practitioners, in fact, is associated with better chances of tumor resectability and patient survival.

The importance of correct and timely treatment of infections in the prevention of gastrointestinal neoplasms is emphasized by two studies included in this Special Issue, carried out by research groups of the Universities of Pittsburgh and Tokyo Hospital [5,6]. The former, by Paragomi et al., focused on the oncologic impact of eradication of *Helicobacter pylori* infection, demonstrating that its successful treatment significantly reduces the risk of gastric cancer [5]. These results are consistent with those of the Japanese study conducted by Ning et al., which highlighted the peculiar significance of eradication of *Helicobacter pylori* infection in neglected subpopulations, such as those with early-onset gastric cancer [6].

Early detection is also crucial in the management of oral squamous cell carcinoma. In this regard, the innovative study by Caprioli et al. is the first that analyzed tumor margins detected on intra-oral ultrasound for predicting histological risk factors [7]. The results are promising and demonstrate the capability of intra-oral ultrasound in predicting the histological staging of the disease, underscoring the importance of this technique in the preoperative management of this neoplasm [7].

Additionally, this collection features studies that emphasize the beneficial role of Selective Serotonin Reuptake Inhibitors, Non-Steroidal Anti-Inflammatory Drugs, and aspirin in reducing colorectal cancer risk [8,9,10]. Despite the importance of these modifiable factors, colorectal cancer risk is also increased in patients affected by Inflammatory Bowel Diseases [11]. In this issue, their relationship in terms of epidemiology and preventive measures is covered in an extensive narrative review, with a particular focus on how the advancing age of this group of patients represents a challenge that will be increasingly encountered in clinical practice, particularly considering the less favourable prognosis of colorectal cancer in this specific group compared to the general population [12].

In addition to primary prevention strategies, this Special Issue also covers secondary prevention approaches, highlighting the importance of early detection and treatment. In fact, one study focused on surveillance strategies for individuals with pancreatic ductal adenocarcinoma associated with the genetic variant *CDKN2A* [13]. The results, after an observational analysis of prospectively collected data from *CDKN2A* carriers who underwent screening for pancreatic cancer, support the latest surveillance recommendations in these individuals that recommend annual surveillance with endoscopic ultrasound and/or magnetic resonance imaging [14].

Furthermore, tertiary prevention, aiming to prevent progression to more advanced stages, plays a crucial role in reducing the burden of these conditions on individuals and society. Investigations into the effects of folic acid supplementation and dietary interventions on colorectal cancer support this concept, as they further highlight the intricate relationship between nutritional factors and epigenetic modifications with the development and progression of gastrointestinal cancers [15]. In this context, adherence to treatment guidelines is also essential, as equitable access to guideline-based care is important for better disease-specific and overall survival outcomes. It is the case of the study revealing disparities in adherence to anal squamous cell carcinoma recommended treatment approach [16]. Following a retrospective analysis of the management of 4740 patients in the US, the findings of this study highlighted the ways in which non-adherence to guidelines is associated with worse disease-specific and overall survival [16]. Furthermore, in the context evaluated, male patients, those with Medicaid insurance, and those with low socioeconomic status are less likely to receive adherent treatments, indicating the need to optimize the delivery of guideline-adherent care to vulnerable populations in order to improve outcomes for all patients with anal squamous cell carcinoma.

In conclusion, this Special Issue provides valuable insights into the epidemiology and prevention of gastrointestinal tract and liver cancers. It highlights the importance of understanding risk factors, implementing targeted interventions, and promoting healthy behaviors to mitigate the increasing burden of these cancers. Addressing modifiable risk factors and adopting preventive strategies can reduce the incidence and mortality of these tumors, ultimately improving global health outcomes.
